# Perineal recurrence presenting as a cutaneous lesion after intersphincteric abdominoperineal resection for low rectal cancer: a case report with implications for surgical planning and postoperative surveillance

**DOI:** 10.1007/s13691-026-00906-x

**Published:** 2026-09-04

**Authors:** Tomohiro Takeda, Shu Chiba, Daisuke Horikawa, Chikayoshi Tani, Yasuhiro Fujiwara, Tomokazu Hoshi, Hideki Yokoo

**Affiliations:** 1 Department of Surgery, Furano Kyokai Hospital, 1-30 Sumiyoshi-cho, Furano, 076-8765 Hokkaido Japan; 2 Division of Gastroenterological and Transplant Surgery, Department of Surgery, Asahikawa Medical University, Asahikawa, Hokkaido Japan

**Keywords:** Rectal cancer, Perineal recurrence, Cutaneous recurrence, Intersphincteric abdominoperineal resection, Postoperative surveillance

## Abstract

A 77-year-old man with low rectal cancer (cT3N0M0) underwent laparoscopic intersphincteric abdominoperineal resection. During the transanal phase, the Lone Star^®^ Retractor System was used, and the anterior wall of the anal canal was slightly injured. Histopathological examination showed T3N0M0 disease with the closest circumferential resection margin measuring 0.6 mm. Computed tomography (CT) performed 3 months after surgery demonstrated a presacral soft-tissue lesion with an enhancing nodule, which remained stable over time and was considered a postoperative change. At 28 months after surgery, perineal bleeding led to the discovery of a friable 3-cm cutaneous tumor in the perineal region, which was locally resected. Histopathology revealed moderately differentiated tubular adenocarcinoma with extensive necrosis. Immunohistochemistry showed cytokeratin (CK) 7 negativity, CK20 positivity, and caudal-type homeobox 2 positivity, consistent with rectal cancer origin. Because the excision margin of the recurrent lesion was negative and CT showed no definite continuity with the presacral lesion, the tumor was regarded as perineal cutaneous recurrence. Although rare, perineal recurrence can occur after rectal cancer surgery. This case highlights the importance of margin-oriented surgical planning and suggests that careful perineal inspection may be considered during postoperative surveillance.

## Introduction

Perineal recurrence after rectal cancer surgery is rare. Possible mechanisms include extension of pelvic local recurrence, implantation of exfoliated tumor cells associated with surgical manipulation, and true cutaneous metastasis. In addition to the relative rarity of cutaneous metastasis from colorectal cancer, recurrence presenting as a perineal or perianal cutaneous lesion after rectal cancer surgery has been reported only sporadically. Herein, we report a case of perineal recurrence after intersphincteric abdominoperineal resection (APR) for low rectal cancer, with discussion of the possible mechanism of recurrence and the importance of perineal examination during postoperative surveillance.

## Case presentation

A 77-year-old man with a history of atrial fibrillation was referred to our department because routine blood testing revealed severe anemia with a hemoglobin level of 6.5 g/dL. On digital rectal examination, a mass was palpable in the lower rectum. His height was 145 cm, weight was 50 kg, and body mass index was 23.8 kg/m². Serum tumor markers were within the normal range (carcinoembryonic antigen (CEA), 1.51 ng/mL; carbohydrate antigen 19 − 9, 3.43 U/mL).

**Fig. 1:**
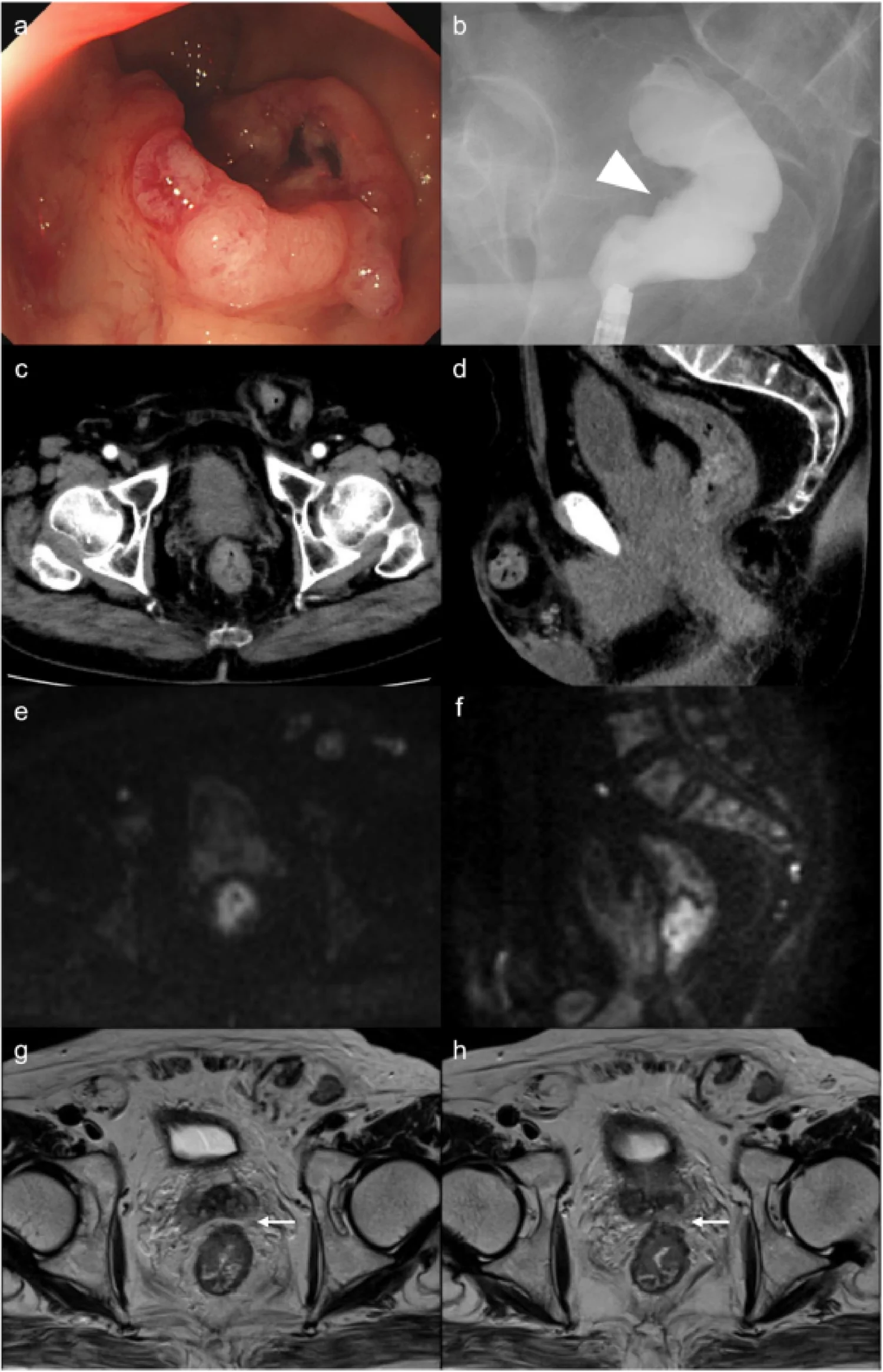
Preoperative endoscopic and radiologic findings. Colonoscopy showed a type 2 tumor in the lower rectum, 3 cm from the dentate line (**a**). Gastrografin enema showed irregular deformity of the anterior wall of the lower rectum (arrowhead) (**b**). Contrast-enhanced computed tomography (CT) showed irregular wall thickening with enhancement in the lower rectum (**c**, **d**). Magnetic resonance imaging (MRI) showed a high signal intensity area on diffusion-weighted imaging corresponding to the lesion seen on CT (**e**, **f**). Axial T2-weighted MRI showing partial loss of the muscularis propria on the right anterior wall and direct tumor abutment to the perirectal fat, without definite invasion of the external anal sphincter or levator ani muscle. Arrows indicate the site of closest proximity to the mesorectal fascia, suggesting a potentially close circumferential resection margin (**g**, **h**)

Colonoscopy revealed a type 2 tumor on the anterior wall of the lower rectum, 3 cm from the dentate line. Gastrografin enema examination showed deformity of the rectal wall at the same site (Fig. 1a, b). Computed tomography (CT) and magnetic resonance imaging (MRI) demonstrated an anteriorly located low rectal tumor with irregular wall thickening, contrast enhancement, and high signal intensity on diffusion-weighted imaging (Fig. 1c–f). Axial T2-weighted imaging showed partial loss of the muscularis propria on the right anterior wall and direct abutment to the perirectal fat, without definite invasion of the external anal sphincter or levator ani muscle. Although the tumor appeared close to the mesorectal fascia, MRI did not show definite mesorectal fascia involvement or raise clear preoperative concern for circumferential resection margin involvement (Fig. 1g, h).

The lesion was diagnosed as low rectal cancer (well-differentiated tubular adenocarcinoma, cT3N0M0). Based on the absence of definite external sphincter or levator ani invasion on preoperative MRI, intersphincteric APR was considered oncologically acceptable at the time of surgery.

Laparoscopic intersphincteric APR was planned and performed. Total mesorectal excision was first carried out laparoscopically. Although dissection was advanced beyond the tumor, it could be extended only to the entrance of the anal canal from the abdominal side. The transanal phase was then performed as planned to secure the distal margin. The transanal phase was initiated using the Lone Star^®^ Retractor System (CooperSurgical, Trumbull, CT, USA). After placement of the retractor, the rectal lumen was closed with a purse-string suture at the mucosal level 2 cm distal to the lower edge of the tumor. Full-thickness transection of the anal canal was then performed proximal to the dentate line, and dissection was continued in the intersphincteric plane to connect with the abdominal dissection plane. During the post-closure transanal dissection, the anterior wall of the anal canal was slightly injured; however, no obvious tumor exposure or fecal spillage was recognized intraoperatively. The levator ani muscle, anal sphincter, and anal mucosa were closed separately. The specimen was extracted through a small laparotomy, and a permanent sigmoid colostomy was created.

**Fig. 2:**
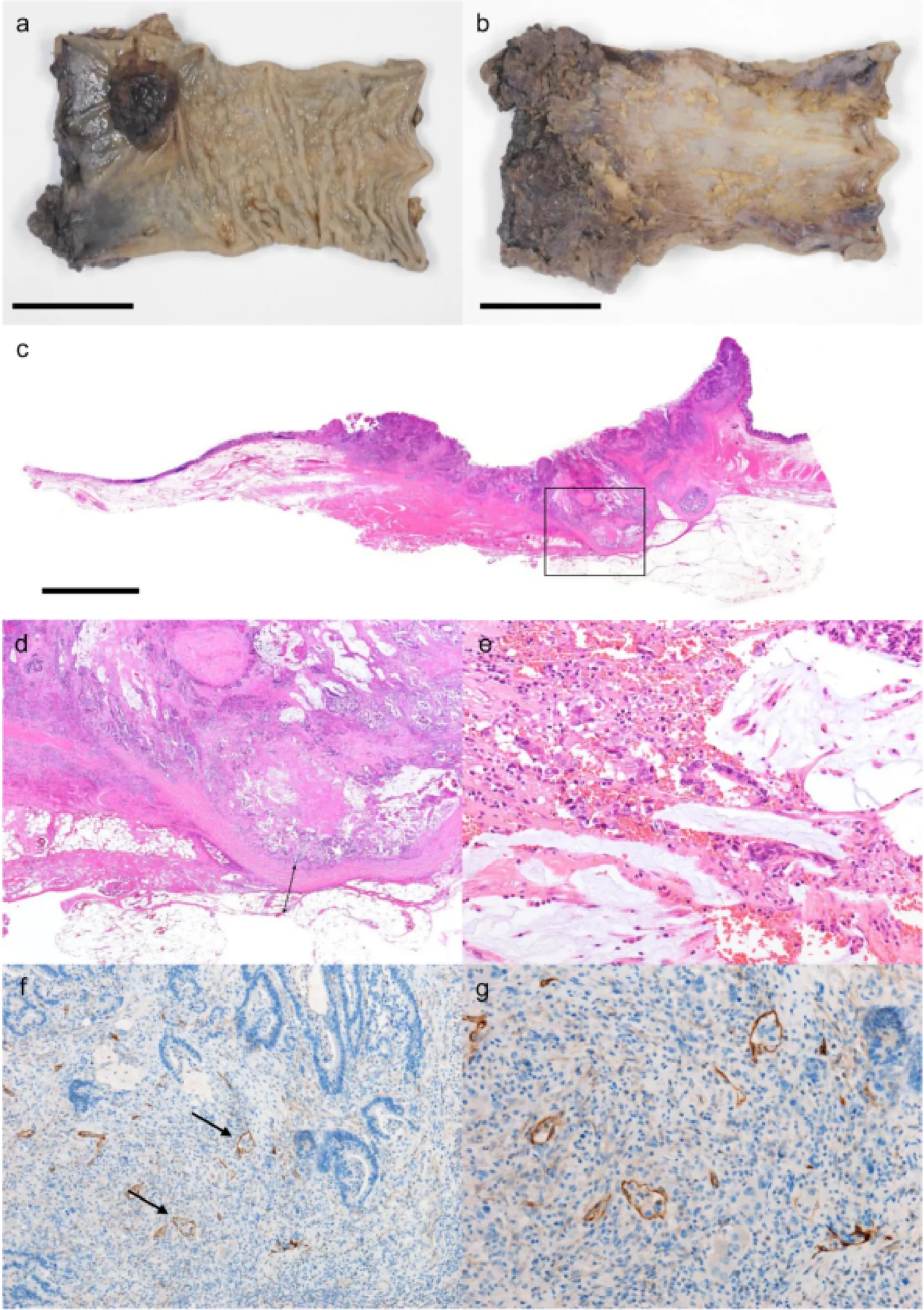
Macroscopic and histopathological findings of the primary rectal cancer. The resected specimen showed a type 2 lesion measuring 45 × 28 mm in the lower rectum (**a**, **b**; scale bar = 50 mm). Histopathological examination showed well- to moderately differentiated tubular adenocarcinoma with a mucinous component at the invasive front. The tumor invaded beyond the muscularis propria. The closest circumferential resection margin (CRM), measuring 0.6 mm, is indicated in panels c and d. The boxed area in panel **c** is enlarged in panel **d**, where the double-headed arrow indicates the site of the closest CRM. Panel **e** shows the histological findings at higher magnification. Lymphatic invasion was identified on D2-40 staining in panels f and g, and the arrows in panel f indicate the positive foci. **c**-**e**: H&E staining (**c**, low-power view, scale bar = 5 mm; **d**, ×2; **e**, ×20). **f**, **g**: D2-40 staining (**f**, ×10; **g**, ×20)

Histopathological examination revealed a type 2 tumor measuring 45 × 28 mm, composed predominantly of well-differentiated tubular adenocarcinoma with moderately differentiated and mucinous components. The pathological stage was T3N0M0, corresponding to Stage IIA. The closest CRM was 0.6 mm, and lymphatic invasion was identified on D2-40 staining (Fig. 2a–g).

Seven months after the initial operation, the patient underwent left colectomy and transverse colostomy for perforation of the stomal bowel. One year after the initial operation, he also underwent repair of an incarcerated left inguinal hernia by the Lichtenstein method.

**Fig. 3:**
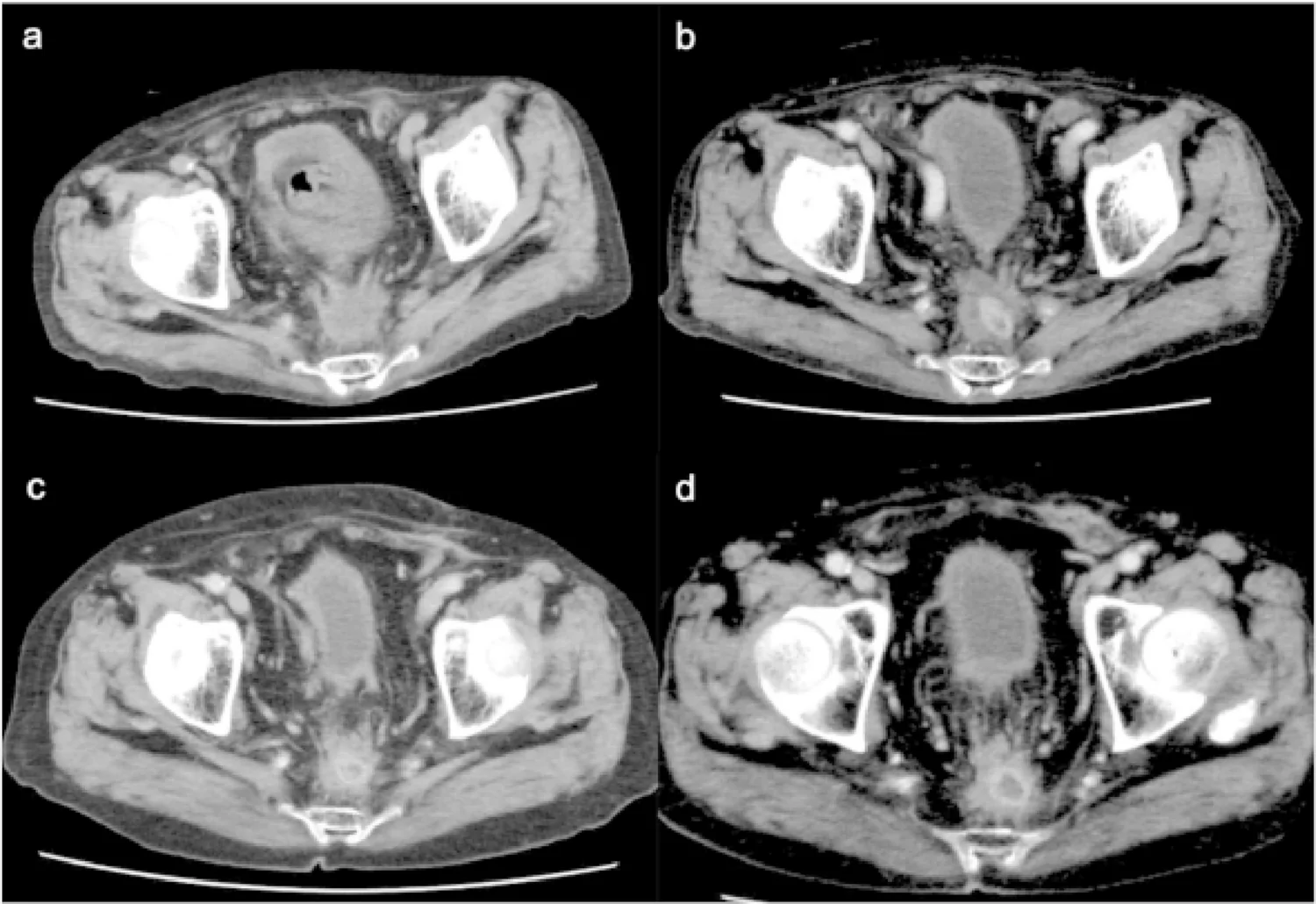
Chronological changes in the presacral soft-tissue lesion with an enhancing nodule after surgery. Plain CT obtained 3 months after surgery is shown in panel **a**. Contrast-enhanced CT obtained 12, 24, and 27 months after surgery is shown in panels **b**, **c**, and **d**, respectively

CT performed 3 months after surgery demonstrated a presacral soft-tissue lesion with an enhancing nodule. Because it did not increase in size over time, it was regarded as a postoperative change and was followed conservatively (Fig. 3a–d). Perineal inspection had not been performed because the patient had no perineal symptoms.

**Fig. 4:**
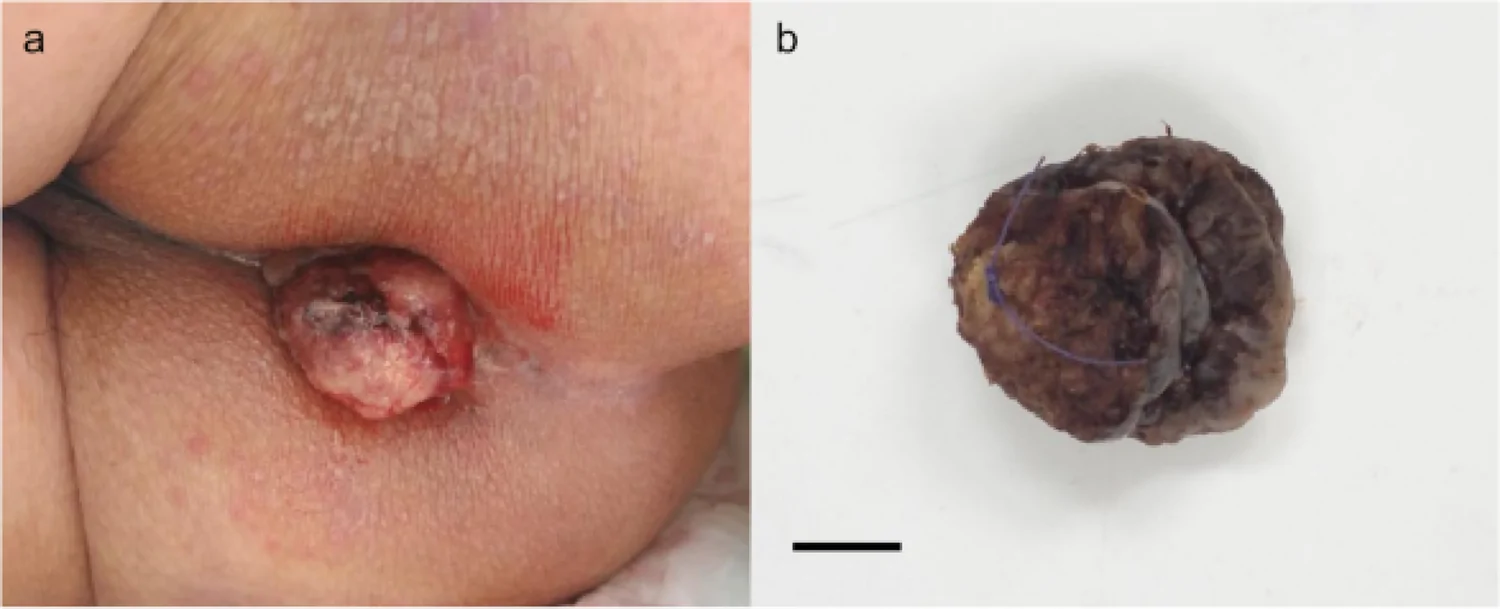
Macroscopic findings of the perineal cutaneous tumor. The gross appearance of the perineal cutaneous tumor is shown in panel **a**. The resected specimen is shown in panel **b**; the surface with the attached suture corresponds to the surgical dissection plane (scale bar = 10 mm)

**Fig. 5:**
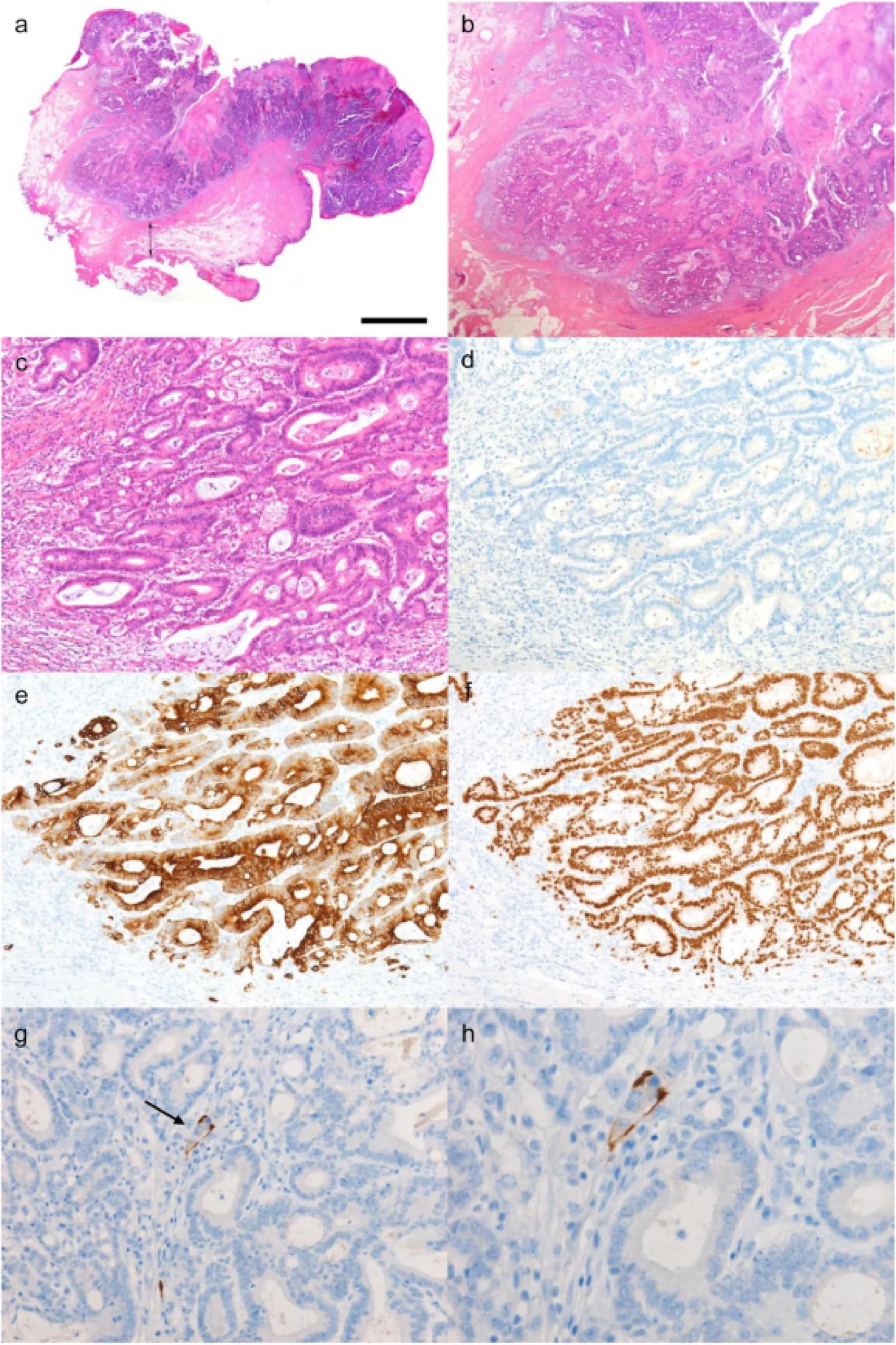
Histopathological and immunohistochemical findings of the perineal cutaneous tumor. Ulcer formation was present in the epidermis, and moderately to well-differentiated adenocarcinoma measuring 28 × 13 mm was identified mainly in the dermis with focal extension into the subcutaneous tissue (**a**-**c**). The excision margin was negative, and the minimum distance to the margin was 2.7 mm (**a**). Immunohistochemically, the tumor cells were negative for cytokeratin (CK) 7 and positive for CK20 and caudal-type homeobox 2 (CDX2), supporting recurrence of rectal adenocarcinoma (**d**-**f**). Lymphatic invasion was identified on D2-40 staining (**g**, **h**), whereas venous invasion was not evident. a-c: H&E staining (a, low-power view, scale bar = 5 mm; **b**, ×4; **c**, ×10). **d**-**f**: immunohistochemical staining for CK7, CK20, and CDX2, respectively, each at ×10. **g**, **h**: D2-40 staining showing lymphatic invasion (**g**, ×10; **h**, ×40); the arrow in panel **g** indicates the positive focus, and panel h shows a higher-magnification view

Twenty-eight months after the initial surgery, the patient was hospitalized for low back pain. Perineal bleeding during this admission led to identification of a friable 3-cm cutaneous tumor in the perineal region. The tumor was locally excised under spinal anesthesia (Fig. 4a, b). No obvious invasion into the sphincter muscle was observed. Histopathological examination revealed moderately differentiated tubular adenocarcinoma with extensive necrosis. The lesion involved the dermis and focally extended into subcutaneous tissue, and the excision margin was negative with a minimum distance of 2.7 mm. Lymphatic invasion was identified, whereas venous invasion was not evident. Immunohistochemistry showed cytokeratin (CK) 7 negativity, CK20 positivity, and caudal-type homeobox 2 (CDX2) positivity (Fig. 5a–f), consistent with recurrence of rectal adenocarcinoma. Because the resection margin was negative and CT showed no definite continuity between the lesion and the presacral abnormality, the lesion was regarded as perineal cutaneous recurrence.

After local excision of the recurrent lesion, PET-CT was not performed because the patient did not wish to undergo further imaging. Additional systemic chemotherapy was not administered because the patient did not wish to receive chemotherapy, consistent with his preference at the time of primary surgery. The patient was followed clinically, and at 36 months after the initial rectal cancer surgery and 6 months after local excision of the recurrent lesion, there was no evident re-recurrence on physical examination or tumor marker assessment. Follow-up CT performed at that time also showed no evident recurrence or metastasis. Thereafter, continued surveillance with CT every 3 months is planned.

## Discussion

The most important issue raised by this case is not only the rarity of perineal recurrence, but also the oncological adequacy of the initial surgical strategy for a T3 low rectal cancer. Conventional APR allows en bloc removal of the rectum, anal canal, and sphincter complex and is an appropriate procedure for low rectal cancer when invasion of the external sphincter or levator ani muscle is suspected. However, it requires a permanent stoma and is associated with perineal wound complications and long-term perineal symptoms [1]. In contrast, intersphincteric APR is intended to reduce pelvic and perineal morbidity by preserving the external sphincter and pelvic floor and by minimizing the perineal wound [2]. Its indication, however, is limited to cases without invasion of the external sphincter or levator ani, and oncological safety depends on achieving a negative CRM. In appropriately selected patients, there is no clear evidence that intersphincteric resection (ISR) itself increases the risk of local recurrence. Akasu et al. reported an overall 3-year local recurrence rate of 5.7% after ISR for very low rectal adenocarcinoma and emphasized the importance of a negative resection margin, although the recurrence rate was higher in T3 disease [3]. This point is particularly relevant in the present case, because the tumor was pathologically T3 and the final CRM was extremely close. In retrospect, the known oncological risk associated with T3 low rectal cancer should have been weighed more carefully in procedure selection and margin strategy, with greater priority given to securing adequate radial clearance. Furthermore, subsequent reports have indicated that ISR does not lead to an excessive increase in local recurrence when appropriate patient selection is performed [4]; however, the present case suggests that such selection must be especially cautious in T3 disease.

Against this background, the rationale for selecting intersphincteric APR in the present case should be reconsidered. In the present case, intersphincteric APR was selected because of the potential advantages described above over conventional APR. However, the closest circumferential resection margin (CRM) was only 0.6 mm, representing an extremely close margin with potential oncological significance. In retrospect, this extremely close CRM suggests that the preoperative estimation of radial clearance may not have been sufficient, particularly in the setting of a T3 low rectal tumor. Therefore, although the procedure was considered oncologically acceptable at the time of surgery, greater emphasis may have been needed on securing radial clearance rather than on minimizing perineal morbidity. In addition, slight injury to the anterior wall of the anal canal occurred during the transanal phase after luminal closure had been completed. No obvious tumor exposure or fecal spillage was recognized intraoperatively. Nevertheless, given that the primary tumor was located predominantly on the anterior wall, this intraoperative event may still have been relevant to the mechanism of recurrence. Therefore, although implantation cannot be proven, it should be considered alongside close CRM as a plausible contributing factor. The presacral lesion showed little chronological change on CT, and no definite radiological continuity with the recurrent perineal lesion was identified. However, this does not exclude the possibility that both lesions were part of the same local recurrence process. Therefore, implantation-related recurrence and recurrence associated with close CRM should be considered as complementary rather than mutually exclusive mechanisms in the present case. Although recurrence specifically at the site of the Lone Star retractor could not be confirmed, this possibility should be considered. In any case, both meticulous operative technique to minimize possible tumor seeding and margin-oriented surgical planning to secure adequate radial clearance are important.

In addition to the oncological implications of the close CRM, the mechanism of recurrence itself also merits discussion. Possible explanations in this case include true distant cutaneous metastasis, extension of pelvic local recurrence toward the perineum, and local recurrence associated with implantation of exfoliated tumor cells during surgery. Previous reports have described perianal cutaneous recurrence after anterior resection and cutaneous perianal or perineal recurrence occurring at sites corresponding to the hook insertion points of the Lone Star retractor, suggesting that microinjury of the perianal skin and seeding of tumor cells into the operative field may contribute to recurrence [5–10]. In the present case, the recurrent lesion developed as an isolated lesion within the perineal operative field, and no other organ recurrence has been identified. However, implantation-related recurrence should be regarded as one plausible mechanism rather than the sole explanation.

This uncertainty is also reflected in the limited number of previously reported cases. Only a limited number of cases of perineal or perianal cutaneous recurrence after rectal cancer surgery have been reported (Table 1). To facilitate comparison with the present case, cases of implantation into a pre-existing anal fistula and cases with evident distant metastases at recurrence were excluded. These lesions have been described after various rectal procedures and were generally detected without evident distant metastases. Several reports also documented the use of a Lone Star retractor, raising the possibility that implantation of exfoliated tumor cells into microinjured perianal tissue may contribute to this unusual pattern of recurrence. The present case shares these features, with an isolated perineal cutaneous lesion developing 28 months after laparoscopic intersphincteric APR.

**Table 1:** Reported cases of perineal or perianal cutaneous recurrence after rectal cancer surgery, with emphasis on possible implantation-related recurrence

First author/ Year	Age / Sex	Primary tumor / stage	Initial surgery	Use of Lone Star retractor	Interval to recurrence	Distant metastases at recurrence
De Friend, 1992 [5]	NR	Rectal cancer, stage NR	AR using EEA stapler	-	7 months	NR
Reed, 1992 [6]	NR	Rectal cancer, stage NR	AR using EEA stapler	-	4 months	NR
Tranchart, 2008 (case 1) [7]	NR	Rectal cancer, stage NR	AR with coloanal anastomosis	+	NR	-
Tranchart, 2008 (case 2) [7]	NR	Rectal cancer, stage NR	AR with coloanal anastomosis	+	NR	-
Cantos-Pallarés, 2012 [8]	83/ Male	Rectal cancer, stage NR	Hartmann’s operation	+	12 months	-
Hamid, 2017 [9]	75/ Female	Rectal cancer, ypStage II	Lap-ISR	+	14 months	NR
van Lieshout, 2022 [10]	57/ Male	Rectal cancer, cStage I	Local Excision by TAMIS	+	23 months	-
Present case	77/ Male	Rectal cancer, pStage II	Lap intersphincteric APR	+	28 months	-

AR, anterior resection; EEA, End to End Anastomosis; ISR, intersphincteric resection; Lap, Laparoscopic; NR, Not Reported; TAMIS, Transanal minimally invasive surgery

Cases of implantation into a pre-existing anal fistula and cases with evident distant metastases at recurrence were excluded

Perineal cutaneous recurrence after rectal cancer surgery is rare [11]. In a review of 100,453 cases, 77 cases of cutaneous metastasis were identified, of which 14.2% originated from gastrointestinal malignancies [12]. Such lesions are often regarded as manifestations of advanced systemic disease and are generally associated with a poor prognosis [13]. However, a few cases have been reported in which a solitary lesion was detected without concomitant visceral metastasis [5–10]. Therefore, a lesion such as that in the present case, which developed in isolation near the original perineal operative field after curative resection and without clear evidence of metastasis to other organs, represents an unusual clinical presentation.

Another important clinical implication of this case is the importance of perineal observation during postoperative surveillance. The aim of surveillance after colorectal cancer surgery is early detection of treatable recurrence. Although classical reviews have recommended physical examination in addition to CEA testing and endoscopy, the evidence directly supporting the effectiveness of physical examination alone has been limited. Kodeda et al. reported that annual clinical examination alone was insufficient to detect asymptomatic local recurrence after rectal cancer surgery [14].

In the present case, imaging studies were continued after permanent stoma creation, but the perineal lesion was recognized only after the onset of bleeding symptoms. Although direct evidence that perineal observation is more likely to be overlooked in patients with a permanent stoma is lacking, this case suggests that perineal lesions that are difficult to detect by imaging alone may occur. More importantly, the case also highlights the need for careful preoperative staging, appropriate patient selection, and margin-oriented surgical planning in low rectal cancer, particularly in T3 disease. After rectal amputation, the perineum remains part of the original operative field and may be affected by local recurrence, implantation, and wound-related lesions. Therefore, postoperative surveillance may also include careful visual inspection and palpation of the perineum, in addition to tumor marker and imaging assessment. However, this observation should be interpreted cautiously, because the present case alone cannot determine whether routine perineal examination would alter clinical outcomes. Moreover, follow-up after excision of the recurrent lesion remains short, and longer observation is required to determine the long-term oncological outcome.

Taken together, this case suggests that, in low rectal cancer, especially T3 disease, the oncological adequacy of the initial operative strategy should be weighed carefully against the potential benefits of reduced perineal morbidity. In addition, postoperative surveillance should remain attentive to the perineal operative field.

## Data Availability

No datasets were generated or analyzed during the current study.

## Abbreviations

APR: Abdominoperineal resection
CEA: Carcinoembryonic antigen
CT: Computed tomography
MRI: Magnetic resonance imaging
CRM: Circumferential resection margin
CK: Cytokeratin
CDX: Caudal-type homeobox 2
ISR: Intersphincteric resection
